# Color Stability and Micro-Hardness of Bulk-Fill Composite Materials after Exposure to Common Beverages

**DOI:** 10.3390/ma13030787

**Published:** 2020-02-09

**Authors:** Nora Bahbishi, Waad Mzain, Bayan Badeeb, Hani M. Nassar

**Affiliations:** 1 Faculty of Dentistry, King Abdulaziz University, P.O. Box 80209, Jeddah 21589, Saudi Arabia; nbahbishi0001@stu.kau.edu.sa (N.B.); wmzain@stu.kau.edu.sa (W.M.); bbadeeb@stu.kau.edu.sa (B.B.); 2 Department of Restorative Dentistry, Faculty of Dentistry, King Abdulaziz University, P.O. Box 80209, Jeddah 21589, Saudi Arabia

**Keywords:** Bulk-Fill, resin composite, color stability, micro-hardness

## Abstract

Objectives: To assess the color stability and surface microhardness of Bulk-Fill composite materials available in the Saudi Arabia market. Methods: Five composite materials (Filtek Z350, Filtek Bulk-Fill, Tetric N-Ceram Bulk-Fill, Sonic Fill 2, and SDR) were investigated. Samples (*n* = 20; 10 mm in diameter and 2 mm in thickness) were fabricated using a stainless-steel mold and were immersed in tea, coffee, berry juice, and distilled water (control). Baseline (T_0_) shades of specimens were recorded using a spectrophotometer and after 10 (T_1_), 30 (T_2_), 60 (T_3_), and 90 days (T_4_) of immersion. Measurements were obtained against a black background and CIE L*a*b* data was used to calculate ΔE for each group. Vickers microhardness values were obtained at T_0_ and T_4_. Data was analyzed using mixed model repeated measure ANOVA at 0.05 significance level. Results: Time, material, and solution effects have statistically significant effect on ΔE. Tea was the most staining solution. Z350 was associated with the highest ΔE values while SDR showed the lowest values. No other materials showed significant difference between each other. Solutions were statistically different from each other. All materials were different from each other regarding microhardness. Conclusion: Bulk-Fill materials showed more color stability but lower microhardness values compared to universal resin control.

## 1. Introduction

Among the different direct restorative dental materials, composite resin is considered the first choice to be used nowadays [1,2]. This is due to many factors including superior esthetics, bonding to tooth structure, and conservative tooth preparation [1,3,4]. However, the bonding procedure and the application of the resin composite material is time-consuming due to the limited depth of cure of conventional resins [5]. This necessitates the use of an incremental application of universal composites, which are marketed to be used in both the anterior and posterior regions of the oral cavity, in order to maintain adequate degree of conversion which has been reported previously to be very crucial for the longevity of resin composite restorations [6,7]. Low degree of conversion due to undercuring is the primary cause of resin restorations’ failures [3].

Among the recent advances in the resin composite formulations, Bulk-Fill formulations are starting to have an increased attention. In contrast to conventional composites, which require incremental placement, these materials contain more sensitive photoinitiators that allow the depth of cure to reach up to 5 mm while maintaining predictable degree of conversion [8,9]. This would allow dentists to place a single increment in deep lesions without the need for a layering technique expediting the restorative procedure and decreasing the overall chair time [10].

Bulk-Fill resin achieves deep depth of cure by utilizing unique composition. This unique chemistry could affect the properties of the resin composite [11]. Even though Bulk-Fill formulations are mainly considered for posterior applications, maintaining basic esthetic characteristics of the resin is required. Color stability and surface microhardness could affect the survival of composite restorations as well as the dentist’s decision for replacement. Color match and anatomic form (including resistance to abrasion) are important to predict the service of resin materials and are two of the parameters used to evaluate the quality of existing restorations based on the United Stated Public Health System (USPHS) [12].

Composite restorations are subjected to extrinsic stains in the oral cavity from multiple sources including smoking, foods and drinks [13,14,15]. It is important to determine the effects of such staining process on Bulk-Fill composites. Thus, the objective of this study was to evaluate the color stability and surface microhardness of Bulk-Fill resin composite materials available in the Saudi Arabian market after immersion in commonly consumed beverages.

## 2. Materials and Methods

### 2.1. Specimen Preparation

Five types of composite materials: Filtek Z350 (Z350; 3M ESPE, Saint Paul, MN, USA), Filtek Bulk-Fill (FB; 3M ESPE, Saint Paul, MN, USA), Tetric N-Ceram Bulk-Fill (TB; Ivoclar Vivadent, Zurich, Switzerland), Sonic Fill 2 (SF2; Kerr Dental, Orange, CA, USA), and SDR (Dentsply, Konstanz, Germany) were used for the study (Table 1). Twenty discs (10 mm in diameter and 2 mm in thickness) from each resin material were fabricated by placing the material in a stainless-steel mold and light curing for 20 s after placing a mylar strip on either side of the mold and pressing gently to remove excess material using microscopic slides. Light curing was done using an LED curing light (DemiUltra, Kerr Dental, Orange, CA, USA) that was checked frequently for irradiance values to be above 1000 mW/cm^2^ using a digital radiometer (Bluephase Meter II, Ivoclar Vivadent Inc., Amherst, NY, USA). A2 shade was used from all materials except for TB, where IVA shade was used as recommended by the manufacturer.

### 2.2. Staining Procedure

Five specimens from each group were placed in each of the following solutions: tea (15 g of loose tea leaves (Al-Kbous black tea, Alkbous Co., Amman, Jordan) simmered in 1 L of boiling water for 5 min)), coffee (15 g of ground coffee (Kurukahveci, Mehmet Efendi, Istanbul, Turk Mali, Turkey) simmered in 1 L of boiling water for 3 min)), berry juice (200 mL of concentrated berry juice (Towt, Alhassany Trading Est., Makkah, Saudi Arabia) mixed with 1 L of chilled water)), and distilled water (as a control). All specimens were stored in the respective staining solution in an incubator at 37 °C (Memmert, Schwabach, Germany) and solutions were replaced every 2 weeks. This produced a 5 × 4 × 5 factorial design with five “material” levels and four “solution” levels giving 20 groups that were followed over five “time” points.

### 2.3. Color Change Determination

Baseline (T_0_) shades for all specimens were recorded using a spectrophotometer (CE7000A, X-rite, Grand Rapids, MI, USA). Each specimen was placed flat on the holding bracket and an area measuring 8 × 3 mm was measured by the device against a black background. The Commission Internationale d’Eclairage (CIE) L*a*b* system data was obtained and used to calculate the ΔE for each time point based on changes compared to baseline measurements by applying the following formula [16]:

$$\Delta E=\;\sqrt{(L_{\mathrm{post}}-{\;L}_{\mathrm{baseline}}){\;}^{2}+(a_{\mathrm{post}}-{\;a}_{\mathrm{baseline}}){\;}^{2}+\;(b_{\mathrm{post}}-{\;b}_{\mathrm{baseline}}){\;}^{2}}.\;$$

$$=\;\sqrt{\Delta L^{2}+\;\Delta a^{2}+\;\Delta b^{2}}$$

Whereas, “baseline” parameters were recorded at T_0_ and “post” parameters were recorded after T_1_ (10 days of immersion). ΔE Values were averaged to give the mean ΔE values for each group. The same procedure was repeated after 30, 60, and 90 days of immersion to provide shade changes (ΔE) values for T_2_, T_3_ and T_4_; respectively.

### 2.4. Microhardness Measurement

Vickers microhardness values were obtained by testing the same specimens before (T_0_) and after 90 days of immersion (T_4_). For each measurement, three indentations were created in each specimen (*n* = 5 for each group) by applying a 5 Newton continuous load for 20 s in a microhardness tester (Wilson Hardness, Illinois Tool Works Test and Measurement, Shanghai, China). Average Vickers microhardness values were calculated for each group.

### 2.5. Statistical Testing

Mixed model repeated measure analysis of variance (ANOVA) followed by pairwise comparisons using Fisher’s Least Significant Difference (LSD) Test were conducted to test the effect of material, staining solution, and time on ΔE and microhardness values. A statistical software, SPSS Ver. 17 (IBM Inc., Armonk, NY, USA) was utilized at 0.05 significance level.

## 3. Results

### 3.1. Color Change (ΔE)

**Figure 1:**
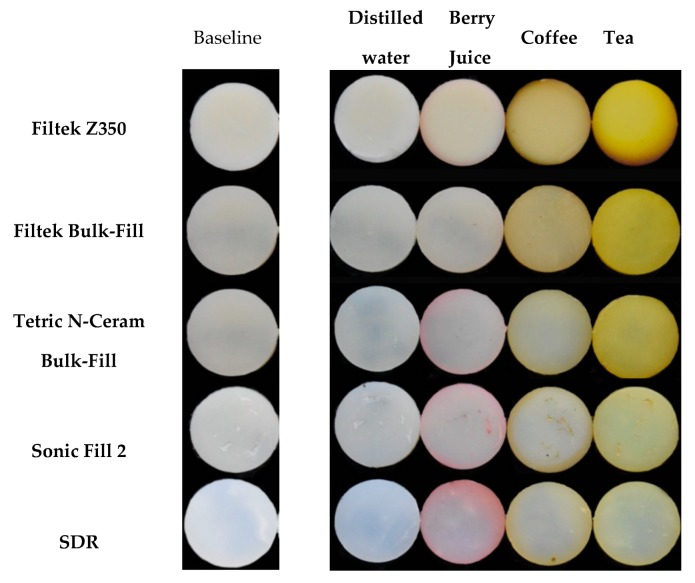
Photographs of specimens from each group of the study at baseline and after 90 days of immersion in the staining solutions.

**Figure 2:**
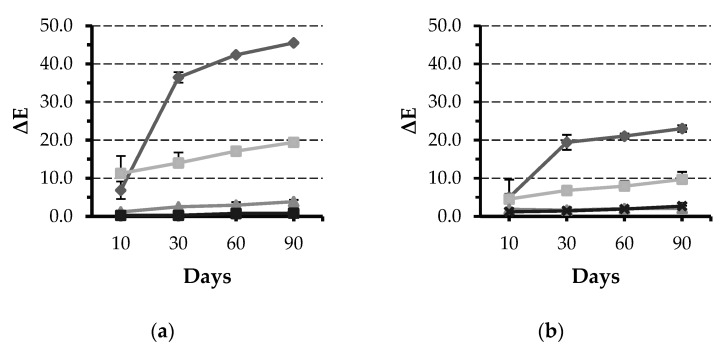

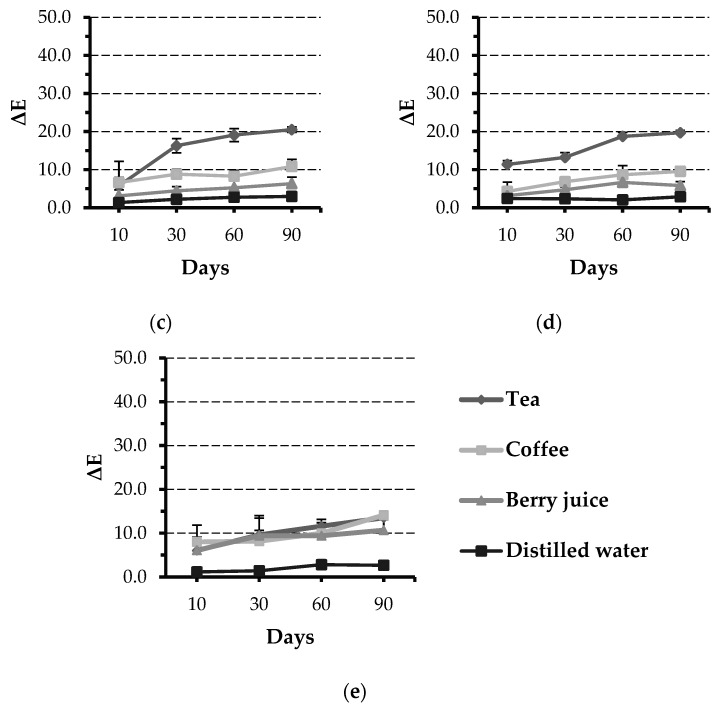
Line graphs showing ΔE values for the five tested materials after immersion in different solutions for 10, 30, 60, and 90 days: (**a**) Filtek Z350, (**b**) Filtek Bulk-Fill, (**c**) Tetric N-Ceram Bulk-Fill, (**d**) Sonic Fill 2, and (**e**) SDR. Error bars represent standard error.

All specimens showed visual changes at T_4_ except in the negative control group (Figure 1). Results of mixed model repeated measure ANOVA showed that time, material, and solutions have statistically significant (*p* < 0.001) effect on ΔE. Tea was the solution leading to the most staining across all groups, except SDR, followed by coffee (Figure 2). All solutions were statistically different from each other regarding ΔE values; however, the magnitude of the effect was dependent on the material.

**Figure 3:**
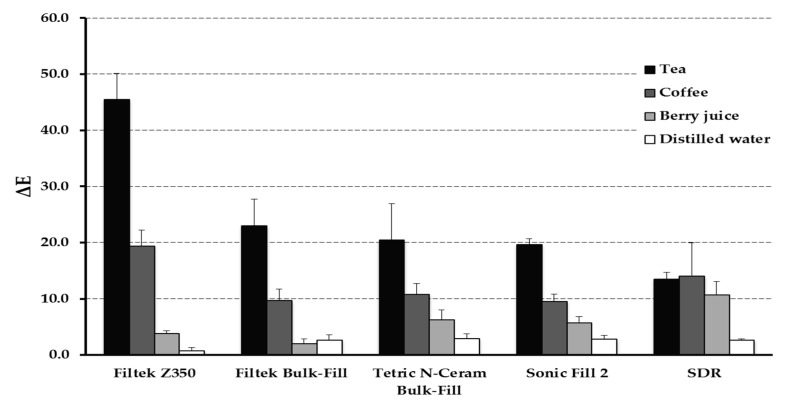
Bar graph showing ΔE values after 90 days of immersion in different solutions. Error bars represent standard error.

Pairwise multiple comparison of “material” effect showed that Z350 is associated with significantly high ΔE values (ΔE at T_4_ = 45.5 ± 4.6; *p* < 0.001) compared to other materials. All Bulk-Fill materials were not statistically different from each other regarding ΔE values (Figure 3). The “time” effect was significant starting T_2_ and the influence increased at T_3_ and T_4_.

### 3.2. Surface Microhardness

Results of mixed model repeated measure ANOVA showed that “time” and “solution” effects were statistically non-significant (*p* > 0.05). However, “material” effect had significant effect on microhardness (*p* < 0.001) with SDR reporting the least microhardness values (VHN = 28.0 ± 2.7) while Z350 was associated with the highest (VHN = 74.2 ± 3.5). Other Bulk-Fill materials had microhardness values in the range of 66.0 and 42.8; yet all of the materials were statistically different from one another (Table 2).

## 4. Discussion

Direct composite restorations are considered a widely spread treatment modality for dental cavities [17]. Advancements in resin formulations have led to the development of new resin formulations that can be considered a viable alternative to dental amalgam [18]. Still, among the major disadvantages of composite resin is the time-consuming nature of the bonding and layering procedure as well as the technique sensitivity associated with the layering method [5,19,20]. Additionally, applying multiple increments can lead to possible incorporation of voids between the layers affecting the integrity of the final restoration [2]. Conventional resin materials required an incremental approach that is necessary to achieve adequate depth of cure for each increment [5,7]. This has led to the development of Bulk-Fill formulations that can be cured in 4 to 5 mm increments [21].

Shade A2 was utilized in this study because it is one of the most prevalent shades in human teeth and commonly used in clinical practice [22]. Filtek Z350 was used as a positive control due to the expansion of data involving this material. In the last couple of years many companies introduced Bulk-Fill variants of their composite brands for posterior restorations. In this in vitro study we investigated some of the widely spread Bulk-Fill formulations in the market. Distilled water was used as a control since it was reported previously to cause no perceivable color change [23].

The CIE L*a*b* system is widely used as an objective modality to judge the colorimetric properties of dental resins [24,25]. This eliminates subjective variability in color perception and allows a standardized approach to determine color changes longitudinally. Changes of lightness of the material (L*) as well as changes in hues across the red-green axis (a*) and yellow-blue axis (b*) can be judged reliably using a spectrophotometer [26,27]. Then, overall shade changes (ΔE) in the material can be calculated using the abovementioned equation. Although, subtle changes in shades can be measured using the spectrophotometer, the significance of these values must be taken into consideration since only ΔE values equal to or larger than 3.7 can be considered visually altered and might require replacement [28].

Of the tested solutions, berry juice has produced the most positive changes in the a* parameter leading to more reddish specimens (Figure 2). However, the majority of the ΔE values can be attributed to changes in the b* (blue-yellow) axis; especially in the tea-exposed groups. This can be clearly seen in Figure 1 where the specimens’ color has shifted to a more yellowish hue except for SDR which appears more reddish since it’s Δa values were slightly higher than the rest of the materials and Δb was the least. Further, a lot of the color changes after 90 days were greater than the 3.7 which can be considered clinically unacceptable (Table 3).

Many of investigations reported that tea was the most staining solution which is in agreement with findings from the current investigation [29,30,31,32]. This was followed by coffee and lastly berry juice produced the lowest values of ΔE (Figure 1 and Figure 2). Only SDR did not follow the same trend. However, a study by Ertas and collaborators reported that there was no significant different between coffee and tea in color change [27]. One reason for these differences could be the methods in which the solutions were prepared, which was not always clear in these investigations, as well as the duration and storage technique. We have decided to continuously store the specimens in the staining solutions throughout the period of the study to simulate long-term exposure to commonly consumed beverages. Based on this investigation and previous ones, it can be concluded that darker solutions can produce more color changes [23]. This can translate clinically into more chances for color changes of composite restorations in patients whose diets contain dark beverages such as tea, coffee, and red wine [33,34,35].

**Figure 4:**
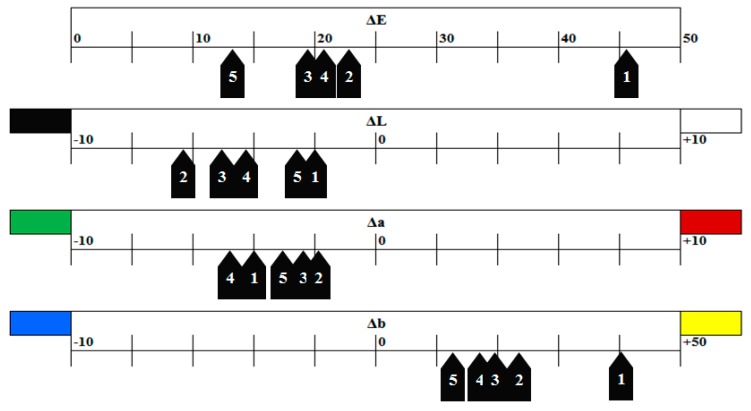
Changes in the CIE Lab parameters for the 5 tested materials after 90 days of immersion in tea solution. (1) Filtek Z350, (2) Filtek Bulk-Fill, (3) Tetric N-Ceram Bulk-Fill, (4) Sonic Fill 2, and (5) SDR. ΔE: overall shade change, ΔL: changes in lightness and darkness, Δa: changes in the red-green axis, Δb: changes in the blue-yellow axis. Please note different scales used for each parameter.

As can be seen in Figure 1 and Table 3, each solution affected the CIE L*a*b* parameters differently. It is beneficial to disclose changes in each parameter rather than mentioning ΔE values alone; since ΔE does not provide the overall picture. This is illustrated more clearly in Figure 4 where changes in the L*a*b* parameters were listed for changes in the groups immersed in tea; which was the solution producing the largest ΔE values across all tested materials (45.5–13.5). Immersing the materials in tea produced a subtle shift towards the greenish end of the a* spectrum. Further, more extreme shift in the b* axis, in the range between +10 and +25 points for Bulk-Fills and +40 for Z350, towards the yellowish end of the spectrum was recorded in specimens stored in tea. This would not have been easily depicted from ΔE alone.

Some investigators have reported more color changes with the increase in composite thickness explaining the liability of Bulk-Fill composite for color changes compared to conventional composites [29,36]. However, the specimen’s setup of this investigation had thickness of 2 mm; this could be a reason for the less color changes in Bulk-Fills in the present study. Further, Tekce and collaborators reported that packable composite had less color change than flowable composite that is usually used in thinner layers [29].

In order to achieve deeper depth of cure, some modifications that could increase the overall translucency of the resin must be made in order to allow deeper penetration of the curing light. [11,37,38]. However, these changes in composition might cause some adverse effects on the color stability of the composite resin. However, the major impact on color stability is caused by water sorption which can be greatly affected by two main parameters: resin matrix to filler ratio and hydrophilicity of the resin matrix. The majority of resin matrix formulations such as Bisphenolglycidyl methacrylate (Bis-GMA) and Urethane dimethacrylate (UDMA) in dental composites are hydrophilic molecules i.e., they attract water with Bis-GMA being slightly more hydrophilic compared to UDMA [39,40]. This will have a direct impact on picking up stains found in beverages. The higher the ratio of resin matrix to fillers within the composite the more water sorption and subsequent color changes will take place [41,42,43]. Additional co-monomers such as Tri-ethylene-glycol-dimethacrylate (TEGDMA) and modified Bis-GMA (Ethoxylated bisphenol-A dimethacrylate; Bis-EMA) and aromatic UDMA (AUDMA) have varying degrees of water affinity. TEGDMA can be considered the least hydrophilic and the aromatic group of AUDMA decreases the water affinity and has a steeper water contact angle compared to UDMA [40]. Overall, studies have shown that UDMA composites tend to have lower water sorption and consequently better color stability compared to Bis-GMA-based resins [39,41,44]. Along with lower filler load, this could potentially the reason for the poor color stability of Z350 in the present investigation. Despite slightly different filler loads, Bulk-Fill formulations in the present study had comparable ΔE values probably owing to complex effects of AUDMA and Bis-EMA in some of them that have lower water affinity compared to Bis-GMA and UDMA.

Values of ΔE continuously increased throughout the study period. However, the analysis of immersion time alone showed that the most pronounced color change occurred after 60 days (Figure 2). This time-dependent increase is in agreement with previous reports where samples exposed to the staining solutions for longer periods were associated with larger shifts in color irrespective of the type of solution used [45,46,47].

In conclusion, It is very difficult to distinguish one solution as the most staining for all Bulk-Fill materials or to deem one more prone to staining in all situations due to the large variations in compositions across composite brands [11]. For each comparison, we suggest considering each of the following factors: (1) material type (conventional, Bulk-Fill, and flowable), (2) solution type (tea, coffee, red wine, berry juice, etc.), (3) solution color (darker solutions affect color more progressively), (4) duration of immersion (longer immersion produces more color changes), and (5) material thickness (thicker layers are more prone to color changes).

As the posterior area of the oral cavity is subjected to high occlusal stresses; Bulk-Fill materials should have sufficient mechanical properties [48]. Generally, the filler size is likely to be positively connected with material properties, such as elastic modulus, strength and hardness [2]. The microhardness of Bulk-Fill composite materials lies in middle between the hybrid resins and flowable composites [10].

According to results from the current investigation, all Bulk-Fill materials tested showed lower microhardness values compared to hybrid composite. Previously, in a study by Ilie and collaborators, the authors reported a similar finding when they tested a group of Bulk-Fill materials [49]. Further, in their study, SDR was associated with lower microhardness values which is in agreement with our results. This is expected since SDR is a flowable Bulk-Fill resin marketed mainly as a dentin replacement and requires a veneering layer of micro-hybrid composite. This was also supported by Sunbul et al. who recommended veneering SDR due to the decrease in microhardness after exposure to food-simulating solvents [50].

As the case with other in-vitro studies, this investigation has some limitations. The storage media did not include saliva due to infection control considerations. This would not simulate the oral environment entirely. In addition, the discs do not fully resemble a restoration from the geometrical perspective. Still, results from the current investigation will be used to formulate designs for clinical experiments in which parameters from this study are considered and can include additional beverages.

## 5. Conclusions

Bulk-Fill composite resins showed lower susceptibility to staining when immersed in tea, coffee, berry juice compared to conventional composite. There were no major differences between Bulk-Fill tested brands regarding color change. Bulk-Fill composites tend to have lower microhardness values that were not affected by immersion in the abovementioned solutions.

## Figures and Tables

**Table 1:** Summary of the resin composite products used in the study.

Material, Abbreviation	Category	Resin Matrix	Main Fillers	Filler Load (wt.%/vol.%)	Photoinitiator	Manufacturer
Filtek Z350 (Z350)	Nano-composite	Bis-GMA, UDMA	Silane-treated ceramic, silica, zirconia	73/56	CQ	3M ESPE, Dental Products, Saint Paul, MN, USA
Filtek Bulk-Fill (FB)	Nano-hybrid Bulk-Fill composite	AUDMA, UDMA, DDDMA	Silane-treated ceramics, silica, zirconia	77/59	CQ	3M ESPE, Dental Products, Saint Paul, MN, USA
Tetric N-Ceram Bulk-Fill (TB)	Nano-hybrid Bulk-Fill composite	Bis-GMA, Bis-EMA, UDMA	Barium glass, silicate glass	81/61	CQ, dibenzoyl germanium derivative (Ivocerin^®^)	Ivoclar Vivadent, Zurich, Switzerland
Sonic Fill 2 (SF2)	Hybrid Bulk-Fill composite	Bis-GMA, TEGDMA, Bis-EMA	Zirconium oxide glass	81/-	Not disclosed	Kerr Dental, Orange, CA, USA
SDR Flow (SDR)	Bulk-Fill flowable composite	Bis-EMA, modified UDMA, TEGDMA	Barium-aluminum-fluorosilicate glass	68/45	CQ	Dentsply; Konstanz, Germany

**Table 2:** Means and standard deviations of Vickers microhardness values of different materials at baseline and after immersion in different solutions for 90 days.

Material	Solution	Vickers Microhardness (Baseline)	Vickers Microhardness (after 90 Days)
Filtek Z350	Tea	74.2 ± 3.6	74.1 ± 5.1
	Coffee	73.8 ± 1.6	75.5 ± 4.8
	Berry juice	74.8 ± 4.6	66.9 ± 4.8
	Distilled water	73.8 ± 4.1	69.4 ± 10.6
Filtek Bulk-Fill	Tea	54.0 ± 3.4	51.1 ± 6.0
	Coffee	55.6 ± 2.0	55.4 ± 4.7
	Berry juice	53.4 ± 3.4	52.9 ± 4.5
	Distilled water	54.0 ± 4.8	46.1 ± 6.0
Tetric N-Ceram Bulk-Fill	Tea	43.2 ± 2.6	47.3 ± 2.4
	Coffee	44.4 ± 3.1	46.3 ± 2.4
	Berry juice	44.0 ± 2.9	43.2 ± 4.7
	Distilled water	42.8 ± 2.6	45.1 ± 2.1
Sonic Fill 2	Tea	63.6 ± 3.1	64.3 ± 3.2
	Coffee	65.4 ± 3.4	67.7 ± 4.2
	Berry juice	66.0 ± 3.9	63.3 ± 2.7
	Distilled water	64.6 ± 0.6	64.3 ± 2.7
SDR	Tea	27.6 ± 2.5	35.9 ± 1.5
	Coffee	28.0 ± 1.9	33.8 ± 5.0
	Berry juice	28.8 ± 2.7	29.1 ± 1.6
	Distilled water	27.5 ± 3.5	33.8 ± 3.5

All materials were significantly different (*p* < 0.001) from each other at baseline and after 90 days immersion. Time (baseline vs. after 90 days) and solution effects were not significant (*p* > 0.05).

**Table 3:** Means and standard deviations of CIE Lab parameters for the materials tested in the four solutions after 90 days of immersion. ΔL: changes in lightness and darkness, Δa: changes in the red-green axis, Δb: changes in the blue–yellow axis, ΔE: overall shade change.

Material	Solution	ΔL	Δa	Δb	ΔE
Filtek Z350	Tea	−6.3 ± 0.5	−2.0 ± 0.6	45.0 ± 4.7	45.5 ± 4.6 *
	Coffee	−9.7 ± 2.2	2.8 ± 1.0	16.6 ± 2.0	19.4 ± 2.8 *
	Berry juice	−2.4 ± 0.5	1.7 ± 0.5	2.4 ± 0.5	3.9 ± 0.5 *
	Distilled water	−0.7 ± 0.5	0.3 ± 0.0	−0.2 ± 0.3	0.9 ± 0.5
Filtek Bulk-Fill	Tea	−2.3 ± 0.8	−4.2 ± 0.7	22.5 ± 4.7	23.0 ± 4.7 *
	Coffee	−4.7 ± 1.3	0.4 ± 0.3	8.4 ± 2.1	9.7 ± 2.0 *
	Berry juice	−1.5 ± 0.9	1.3 ± 0.2	−0.4 ± 0.6	2.1 ± 0.7
	Distilled water	−1.2 ± 0.9	0.4 ± 0.1	−2.3 ± 0.6	2.7 ± 0.9
Tetric N-Ceram Bulk-Fill	Tea	−5.5 ± 1.7	−2.7 ± 0.8	19.6 ± 6.2	19.7 ± 1.0 *
	Coffee	−5.8 ± 0.9	−0.7 ± 0.3	9.1 ± 1.7	9.6 ± 1.3 *
	Berry juice	−4.1 ± 0.9	4.6 ± 1.4	1.3 ± 0.8	5.8 ± 1.1 *
	Distilled water	−2.9 ± 0.7	0.8 ± 0.2	−0.3 ± 0.6	2.9 ± 0.6
Sonic Fill 2	Tea	−4.6 ± 0.7	−5.3 ± 0.7	18.4 ± 1.1	20.5 ± 6.4 *
	Coffee	−5.3 ± 0.8	−1.8 ± 0.3	7.7 ± 1.5	10.8 ± 1.9 *
	Berry juice	−4.2 ± 1.0	3.9 ± 0.7	−0.9 ± 0.7	6.3 ± 1.7 *
	Distilled water	−1.5 ± 0.7	1.3 ± 0.2	−2.0 ± 0.6	3.0 ± 0.7
SDR	Tea	−2.7 ± 1.1	−3.5 ± 0.4	12.8 ± 1.2	13.5 ± 1.1 *
	Coffee	−6.8 ± 8.6	−1.5 ± 0.1	10.8 ± 1.7	14.1 ± 5.9 *
	Berry juice	−3.0 ± 1.2	6.4 ± 1.3	3.0 ± 8.5	10.7 ± 2.4 *
	Distilled water	−2.0 ± 0.0	1.0 ± 1.1	−1.5 ± 0.42	2.7 ± 0.2

* Visually detectable change (mean ΔE ≥ 3.7).

## Abbreviations

AUDMA	Aromatic urethane dimethacrylate
Bis-GMA	Bisphenolglycidyl methacrylate
Bis-EMA	Ethoxylated bisphenol-A dimethacrylate
CQ	Camphorquinone
DDDMA	1,12-Dodecane dimethacrylate
TEGDMA	Tri-ethylene-glycol-dimethacrylate
UDMA	Urethane dimethacrylate
wt.%	weight percentage
vol.%	volume percentage
